# Hirschsprung’s disease in children: a five year experience at a University teaching hospital in northwestern Tanzania

**DOI:** 10.1186/1756-0500-7-410

**Published:** 2014-06-28

**Authors:** Joseph B Mabula, Neema M Kayange, Mange Manyama, Alphonce B Chandika, Peter F Rambau, Phillipo L Chalya

**Affiliations:** 1 Department of Surgery, Catholic University of Health and Allied Sciences-Bugando, Mwanza, Tanzania; 2 Department of Paediatrics, Catholic University of Health and Allied Sciences-Bugando, Mwanza, Tanzania; 3 Department of Anatomy, Catholic University of Health and Allied Sciences-Bugando, Mwanza, Tanzania; 4 Department of Pathology, Catholic University of Health and Allied Sciences-Bugando, Mwanza, Tanzania

**Keywords:** Hirschsprung’s disease, Clinical presentation, Management, Outcome, Tanzania

## Abstract

**Background:**

Hirschsprung’s disease (HD) is the commonest cause of functional intestinal obstruction in children and poses challenges to pediatricians and pediatric surgeons practicing in resource-limited countries. This study describes the clinical characteristics and outcome of management of this disease in our setting and highlights challenges associated with the care of these patients and proffer solutions for improved outcome.

**Methods:**

This was a descriptive prospective study of children aged ≤ 10 years who were histologically diagnosed and treated for HD at our centre between July 2008 and June 2013.

**Results:**

A total of 110 patients (M: F ratio= 3.6:1) with a median age of 24 months were studied. Six (5.5%) patients were in the neonatal period. Sixty-four (58.2%) patients had complete intestinal obstruction whereas 42 (38.2%) and 4 (3.6%) patients had chronic intestinal obstruction and intestinal perforation respectively. No patient had enterocolitis. Constipation (94.5%) was the most common complaints. 109 (99.1%) patients had colostomy prior to the definitive pull-through. The median duration of colostomy before definitive pull-through was 4 months. The majority of patients (67.3%) had short segment of aganglionosis localized to the recto-sigmoid region. The definitive pull-through was performed in 94 (85.5%) patients (Swenson’s pull-through 76 (80.9%), Duhamel’s pull-through (12.8%) and Soave’s pull-through 4 (4.3%) patients). Postoperative complication rate was 47.3%. The median length of hospital stay was 26 days. Patients who developed complications stayed longer in the hospital and this was statistically significant (p <0.001). Mortality rate was 21.8%. The age < 4 weeks, delayed presentation and surgical site infection were the main predictors of mortality (p < 0.001). During the follow-up period, the results of Swenson’s and Duhamel’s pull through procedures were generally good in 87.8% and 42.9% of patients respectively. The result of Soave’s procedures was generally poor in this study.

**Conclusion:**

HD remains the commonest cause of functional intestinal obstruction in children and contributes significantly to high morbidity and mortality in our setting. The majority of patients present late when the disease becomes complicated. Early diagnosis and timely definitive pull through procedure are essential in order to decrease the morbidity and mortality associated with this disease.

## Background

Hirschsprung’s disease, also known as congenital megacolon or congenital colonic aganglionosis, is a developmental disease characterized by absence of ganglion cells in submucosal (Meissner’s) and myenteric (Aurbach’s) plexuses in distal bowel extending proximally for variable distances that result in functional intestinal obstruction caused by dysmotility of the diseased segment [1]. It is one of the most common surgical conditions in the paediatric age group with an incidence of approximately 1 in 5,000 live births [1-3]. Hirschsprung’s disease is caused by the failure of ganglion cells to migrate cephalocaudally through the neural crest during fourth to 12 weeks of gestation, causing an absence of ganglion cells in all or part of the colon [2]. The aganglionic segment usually begins at the anus and extends proximally [4]. Short-segment disease is most common and is confined to the recto-sigmoid region of the colon. Long-segment disease extends past this region and can affect the entire colon. Rarely, the small and large intestines are involved [5].

The cause of Hirschsprung’s disease is multifactorial, and the disease can be familial or develop spontaneously [6]. The disease is more common in boys than girls [7].

The clinical presentation of Hirschsprung’s disease ranges from neonatal intestinal obstruction to chronic progressive constipation in older children. Approximately 80 percent of patients present in the first few months of life with difficult bowel movements, poor feeding, and progressive abdominal distention [7]. In Africa only 20-40% present as neonates, compared to more than 90% in developed countries [8-12]. The diagnosis is mainly by radiographic studies, anorectal manometry and histological examination of rectal wall biopsies [13].

Since its first description in by Harald Hirschsprung, the understanding and the management of the condition has improved greatly. The first successful treatment of the condition was undertaken over six decades ago by Orvar Swenson [14]. The original operation (the Swenson procedure) consisted of freeing the defective distal colon from within the pelvis by careful sharp extra-rectal dissection down to 2 cm above the dentate line and performing an end-to-end anastomosis. Since this first definitive operation was described, many other patients have been treated successfully with other operations including the retro-rectal pull-through of Duhamel and its modifications and the endorectal pull-through of Soave procedure and its various modifications [15-18]. Currently, these procedures are either laparoscopically assisted or accomplished completely via a trans-anal route without abdominal incision [19,20].

In developing countries such as Tanzania, Hirschsprung’s disease poses a diagnostic and therapeutic challenge. Ignorance and poverty on the part of the parents, late presentation with attendant complications, limited access to trained paediatric surgeons and limitation of facilities for prompt diagnosis characterize management of this disease. Hence, multiple stages of management still predominate in sub Saharan Africa [7,8,11,12,21].

In many hospitals in sub-Saharan Africa, particularly the rural hospitals, the facilities and manpower required for extensive neonatal surgery, and placing clinically unstable children under general anesthesia for long hours are not available, hence colostomy which is fast to create is commonly used. Even when the children with Hirschsprung’s disease present in stable clinical state, an initial colostomy is created because it is believed that the atonic proximal segment needed to regain its tone before pull-through and the distal anastomosis needed protective colostomy [7,8,11,12]. However, the morbidities and mortalities associated with colostomy creation are enormous, and this resulted in many centers avoiding its use, and preferring the one stage pull-through [11,12].

Despite the fact that Hirschsprung’s disease in children is prevalent in our environment; little work on this subject has been done in Tanzania and the study area in particular. This is partly due to paucity of local data regarding this condition and lack of community awareness on the importance of early reporting to hospital for early diagnosis and treatment. This study was carried out to determine the clinical characteristics and outcome of management of Hirschsprung’s disease at our center and to highlight challenges associated with the care of these patients and proffer solutions for improved outcome.

## Methods

### Study design and setting

This was a descriptive prospective study of children aged 10 years and below who were histologically diagnosed and treated for Hirschsprung’s disease at Bugando Medical Centre during the 5-year period between July 2008 and June 2013. Bugando Medical Centre is the only tertiary health institution serving the whole of the northwestern part of Tanzania, serving a population of about 13 millions. It is a 1000 bed referral hospital located in Mwanza city in the northwestern Tanzania on the southern border of Lake Victoria. It is also a teaching hospital for the Catholic University of Health and Allied Sciences (CUHAS).

### Study population

The study population included all patients aged 10 years and below who were histologically diagnosed and treated for Hirschsprung’s disease at Bugando Medical Centre during the period of study. Children above 10 years with surgical problems are admitted in the adult surgical wards and therefore were excluded from the study. Recruitment of patients was done at the Accident and Emergency department, in the paediatric medical and surgical wards and thereafter followed up at the paediatric surgical outpatient clinic. Patients who met the inclusion criteria were consecutively enrolled in the study after an informed written consent sought from the parents or guardians.

The diagnosis of Hirschsprung’s disease was made either by barium enema or by a full-thickness rectal biopsy for histopathological examination. Biopsy was also taken from the colostomy edge to rule out long-segment aganglionosis. Relevant preoperative investigations included packed cell volume, serum electrolytes, urea and creatinine, blood grouping and cross-matching. Radiological investigations including X-ray abdomen erect and supine were done in all patients. Barium enema was done is selected patients. Histopathological examination to confirm the diagnosis of Hirschsprung’s disease was done in all the patients.

Preoperatively, all patients who presented with acute intestinal obstruction had intravenous fluids to correct fluid and electrolyte deficits; nasogastric suction and broad-spectrum antibiotic coverage. After resuscitation all patients under general anesthesia were subjected to emergency diverting colostomy to decompress the bowel. Patients who presented with chronic constipation were subjected to elective diverting colostomy also to decompress the colon. The patients thereafter had full thickness rectal biopsy to confirm absence of ganglion cells in both myenteric and sub mucous plexuses. Biopsy was also taken from the edge of colostomy. The results usually came out within a week or two of taking the biopsy. The patients were advised to remain on the ward during this period. Bowel decompression was continued during this time.

Definitive pull through surgery was performed after stabilization of the general condition of the patient. All patients in this study underwent three-staged surgery involving a diverting colostomy, definitive pull-through and colostomy closure. The pull-through operations were performed by a consultant surgeon under general anesthesia. The most commonly performed operations were the Swenson, Soave, and Duhamel procedures. In all these procedures, the aganglionic as well as the dilated proximal bowel segments were resected, and the visibly normal bowel segment was used for the pull-through. Swenson’s operation involved removing the rectum, pulling the healthy ganglionated colon through, and connecting it to the anus [14]. The Soave endorectal pull-through consists of stripping the rectal mucosa with preservation of the rectal muscular cuff. Ganglionated colon is pulled through the muscular cuff and anastomosed just above the dentate line [16]. The Duhamel procedure entails leaving the native rectum in situ and bringing the normally innervated colon behind the rectum with an end-to-side anastomosis 2 cm above the dentate line and joining the two lumens side to side. Because of unavailability of GI stapler for the final anastomosis, we performed a modified Duhamel pull-through procedure using a hand sewn technique as described by Adeniran *et al.*[22]. Histological specimens were obtained from the bowel used for pull-through to ensure that a well ganglionated bowel segment was used.

Postoperatively patients were kept nil orally till return of bowl sounds and at that time nasogastric tubes were removed. IV antibiotics were used for up to one week.

The results of the definitive pull through procedures were graded as good, satisfactory or poor according to the classification suggested by Nielson and Madsen [23], as follows:-

a. *“Good*”, if the patient had no complaints and passed normal stools spontaneously with a maximum interval of 2 days.

b. “*Satisfactory*” if the patient had only minor bowel disorders such as constipation which could be managed by laxatives but never required enemas, occasional diarrhea, occasional slight abdominal distention and occasional soiling.

c. “*Poor*” if there were frequent distention of the abdomen and severe constipation requiring treatment with enemas.

Data on each patient were entered into a pro forma prepared for the study. The study variables collected included age, sex and clinical presentation, type of colostomy done and complications if any, proximal level of aganglionosis, age at definitive pull through, levels of resection, and any complication of definitive pull through. Other information recorded included length of hospitalization, mortality, need for secondary surgery and bowel and sphincteric activities at follow-up. Patients were followed up for a period of twelve months or till death whichever is earlier.

### Statistical data analysis

The statistical data analysis was performed using statistical package for social sciences (SPSS) version 17.0 for Windows (SPSS, Chicago IL, U.S.A). The median + Interquartile Range (IQR) and ranges were calculated for continuous variables whereas proportions and frequency tables were used to summarize categorical variables. Chi-square (*χ*2) test were used to test for the significance of association between the independent (predictor) and dependent (outcome) variables in the categorical variables. The level of significance was considered as p < 0.05. Study variable that was found to be statistically significant in univariate analysis were subjected to multivariate logistic regression analysis. Multivariate logistic regression analysis was used to determine predictor variables that predict the postoperative complications, hospital stay and mortality.

### Ethical consideration

Ethical approval to conduct the study was obtained from the CUHAS-Bugando/BMC joint institutional ethic review committee before the commencement of the study. Informed consent was sought from each patient’s next of kin/parents before being enrolled into the study.

## Results

### Patient’s characteristics

**Figure 1:**
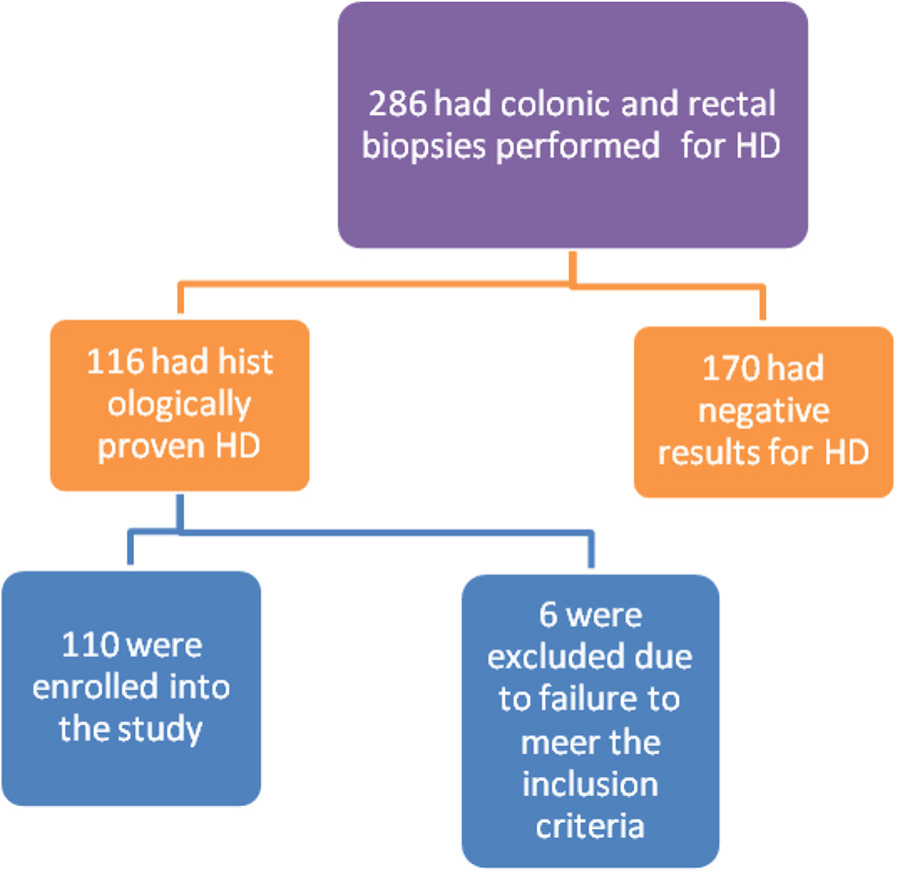
Flow chart of patients.

During the study period, a total of 286 had colonic and rectal biopsies performed for Hirschsprung’s disease. Of these, only 116 patients had histologically confirmed cases of Hirschsprung’s disease. Out of 116 cases of Hirschsprung’s disease, six were excluded from the study due to failure to meet the inclusion criteria and incomplete data. Thus, 110 patients were enrolled into the study (Figure 1). The age of patients at presentation ranged from 7 days to 10 years with a median age of 24 months (+IQR of 22 to 26 months). Only six (5.5%) patients were in the neonatal period. Eighty-six (78.2%) were males and 24 (21.8%) females, with a male to female ratio of 3.6: 1.

### Clinical presentation among patients with Hirschsprung’s disease

At presentation, sixty-four (58.2%) patients presented with complete intestinal obstruction while 42 (38.2%) presented with chronic intestinal obstruction and 4 (3.6%) patients had intestinal perforation. There was no patient with enterocolitis. Constipation and abdominal distention were the most common presenting complaints in 94.5% and 92.7% of cases, respectively (Table 1). There was just one (0.9%) patient with Down’s syndrome.

**Table 1:** Distribution of patients according to clinical presentation

**Clinical presentation**	**Frequency**	**Percentages**
Constipation	104	94.5
Abdominal distention	102	92.7
Wasting	73	66.4
Failure to thrive	68	61.8
Visible colonic peristalsis	25	22.7
Vomiting	25	22.7
Growth retardation	20	18.2
Fever	12	10.9
Spurious diarrhea	7	6.4

### Laboratory, radiological and histopathological investigations

Complete Blood Count, Hemoglobin levels and cross-matching were done in all patients. More than three quarter of the patients had Hemoglobin levels less than 10.0 gm/dl. Serum electrolytes revealed hypokalaemia and hyponatraemia in 64 and 45 patients respectively. Plain abdominal x-rays (erect/supine) done in all patients revealed multiple dilated loops of bowel with significant air-fluid levels in erect films in 68 (61.8%) patients. Free air under the right dome of diaphragm (pneumoperitonium) was seen in four (3.6%) patients who had bowel perforation. Barium enema was done in 34 (30.9%) patients and classically demonstrated the transition zone. Histopathological examination confirmed the diagnosis of Hirschsprung’s disease in all the patients.

### Surgical treatment among patients with Hirschsprung’s disease

**Figure 2:**
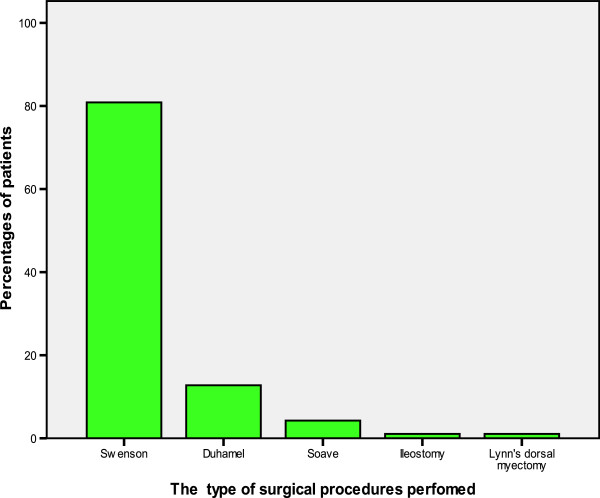
Distribution of patients according to the type of surgical procedure performed.

One hundred and nine (99.1%) patients in this study had colostomy prior to the pull-through. One (0.1%) patient had ileostomy due to total colonic aganglionosis. Seventy-five (68.8%) of the colostomies were placed in the right transverse colon (transverse colostomy) and the remaining 34 (31.2%) in the sigmoid colon (sigmoid colostomy). Eighty-two (74.5%) of the colostomies were loop colostomy while 28 (24.5%) were double barreled. Four (3.6%) patients who had sigmoid perforations underwent double barreled sigmoid colostomy. The median age at formation of colostomy was six months (+IQR of 4 to 8 months) with a range of 12 days to 8 years. The duration of the colostomy before definitive pull-through varied from one to 16 months with a median duration of 4 months (+IQR of 2 to 6 months).The definitive pull-through was performed in 94 (85.5%) patients. Out of 94 patients who had definitive pull-through, 76 (80.9%) underwent Swenson’s pull-through and the remaining twelve (12.8%) and four (4.3%) patients underwent Duhamel’s and Soave’s pull-through procedures, respectively. In one (1.1%) patient who had an ultra-short segment of the disease, Lynn’s dorsal myectomy was done because of the ease of application. Ileostomy was performed after total colectomy in one (1.1%) patient who had total colonic involvement (Figure 2).

The median age of patients at definitive pull-through was 14 months (+IQR of 12 to 16 months) with a range of two months to 10 years. Regarding the level of aganglionosis, seventy-four (67.3%) patients had short segment localized to the recto-sigmoid region, eighteen (16.4%) had long segment, one (0.9%) had total colonic segment and ultra-short segment each, respectively. The level of aganglionosis was not established in sixteen (14.5%) patients. There were no cases of total intestinal involvement. A total of 78 patients had their colostomies closed at the end of study period and the remaining 16 colostomies were not yet closed; 13 because of death before colostomy closure and 3 were still waiting for definitive pull through. The time interval from definitive pull through to colostomy closure ranged from 1 month to 5 months with a mean of 2 months (+IQR of 1 to 4 months).

### Treatment outcome

Of the 110 patients, 52 developed postoperative complications giving a complication rate of 47.3%. These were classified as complications related to colostomy construction, complications related to definitive pull through, complications related to colostomy closure and general postoperative complications as shown in Table 2.

**Table 2:** Distribution of patients according to post-operative complications (N = 52)

**Post operative complication**	**Frequency**	**Percentages**
**Colostomy-related complications (N = 49)**		
• *Skin excoriations*	23	46.9
• *Colostomy prolapse*	21	42.9
• *Non-function*	9	18.4
• *Colostomy retraction*	8	16.3
• *Intestinal obstruction*	2	4.1
• *Parastomal hernia*	2	4.1
**Complications related to definitive pull through (N = 37)**		
• *Surgical site infections*	12	32.4
• *Soiling*	10	27.0
• *Wound dehiscence*	5	13.5
• *Paralytic ileus*	5	13.5
• *Anastomotic leak*	2	5.4
• *Intra-abdominal sepsis*	2	5.4
• *Fecal incontinency*	2	5.4
**Complications related to colostomy closure (N = 34)**		
• *Surgical site infections*	11	32.4
• *Wound gaping*	6	17.6
• *Intestinal obstruction*	2	5.9
• *Ugly incisional scar*	2	5.9
**General complications (N = 20)**		
• *Pneumonia*	4	20.0
• *Urinary tract infection*	3	15.0
• *Septicemia*	3	15.0

The length of hospital stay after colostomy formation ranged from 3 days to 14 days with a median duration of 6 days (+IQR of 4 to 8 days). The overall median duration of hospitalization following definitive pull through was 26 (+IQR of 24 to 28) days (range 1 day to 34 days). The overall length of hospital stay following colostomy closure ranged from 1 to 10 days with a median of 5 days (+IQR of 3 to 7 days). Patients who developed complications stayed longer in the hospital and this was statistically significant (p <0.001).

In this study, a total of 24 patients died giving mortality rate of 21.8%. Out of the 24 deaths, eight deaths were related to colostomy formation, thirteen deaths were due to complications related to definitive treatment and three deaths were related to colostomy closure. According to multivariate logistic regression, the age < 4 weeks (OR = 4.6, 95% C.I. (1.6- 8.9), *p* = 0.002), delayed presentation (OR = 16.3, 95% CI (7.4- 18.9), *p =* 0.011), surgical site infection (OR =3.5, 95% CI (1.4-4.6), *p =* 0.027) were the main predictors of mortality.

### Follow-up of patients

Out of the 86 survivors who had definitive pull-through, eighty-two (95.3%) patients were discharged well and the remaining three (3.5%) patients were discharged against medical advice. One (1.2%) patient among the survivors had permanent ileostomy. The follow up period ranged from 3 to 24 months with a median of 8 months (+IQR of 6 to 10 months). Using classification suggested by Nielson and Madsen [23], out of 82 patients who were discharged well, 68 (82.9%) had good results, 10 (12.2%) had satisfactory results and 4 (4.9%) had poor results. The results of pull through procedures among 82 survivors are shown in Table 3.

**Table 3:** Distribution of patients according to results of pull through procedures among 82 survivors

**Pull through procedure**	**Results of pull through procedures**			**Total**
	**Good**	**Satisfactory**	**Poor**	
Swenson’s procedure	65 (87.8)	7 (9.5)	2 (2.7)	74 (100)
Duhamel’s procedure	3 (42.9)	3 (42.9)	1 (14.3)	7 (100)
Soave’s procedure	-	-	1 (100)	1 (100)
**Total**	**68 (82.9)**	**10 (12.2)**	**4 (4.9)**	**82 (100.0)**

## Discussion

Hirschsprung’s disease (HD) is a common cause of intestinal obstruction in children. In this study, the majority of patients presented after the first year of life and only 5.5% of patients were diagnosed during the neonatal period. This agrees with other reports in Africa [8-12], but the picture is at variance with the presentation in developed countries where more than 90% of patients present early, largely during the neonatal period [24].

This study showed that males were more affected than females with a male to female ratio of 3.6:1 which is comparable to the global ratio of 2.9: 1 to 4.5: 1 [8-10,12,21]. However, an exceptionally high male to female ratio of 22.3: 1 was reported by Ziad *et al*. [23]. The reason for this male predominance is unclear and warrants further investigation.

The clinical presentation of Hirschsprung’s disease in our patients is not different from those in other studies [8,11,12], with constipation and abdominal distention being common to all the patients. As reported by many authors in developing countries [7,8,11,12,21], majority of patients in the present study presented late in poor general condition. In developing countries like ours where over 60% of the populace cannot afford hospital treatment, patients seek hospitalization only when they had developed irreversible intestinal obstruction, abdominal distension or enterocolitis [24]. This is normally when the habitual enema can no longer afford anymore relieve. This observation is reflected in our study where more than fifty percent of patients presented late with acute intestinal obstruction and bowel perforations. Late reporting of patients to hospital in the most developing countries like Tanzania may be attributable to ignorance and poverty [7,8,11,12,21]. This delayed presentation increases morbidity and mortality many-folds, as is evident from our results. We could not establish the reasons for late presentation in this study.

Hirschsprung’s disease has been reported to be associated with neurologic, cardiovascular, urologic, and gastrointestinal abnormalities. Down syndrome (trisomy 21) is the most common chromosomal abnormality associated with the disease, accounting for approximately 10 percent of patients [12,25]. In this study, there was one patient with Down’s syndrome among the hospitalized ones. The diagnosis of Hirschsprung’s disease may be difficult in this group of patients, since the common symptom of constipation in Down’s syndrome could have different reasons such as the reduction of thyroid activity, general myasthenia or hypophrenia [25]. The lower incidence of Down’s syndrome among the patients with Hirschsprung’s disease in this study may be due to the difference of the incidence of Down’s syndrome in general population. The low rate of Down’s syndrome in this study may also be due to pre-hospital mortality from associated malformations e.g. cardiac malformations.

Rectal biopsy remains the gold standard in confirming HD and it shows absence of ganglion cells and presence of hypertrophied nerve fibers [26]. This could be achieved either by a suction biopsy or a full thickness biopsy [27]. The suction biopsy is advantageous because it needs no anesthesia, can be done as a clinic procedure and in good hands has a low false positive rate [26,27]. However, suction biopsy was not available in our center hence the need for general anesthesia in our patients as the full thickness biopsy can only be done under general anesthesia. Barium enema may help with diagnosis but the classical transition zone may not be obvious in the first three months [28]. In our study, barium enema was performed in only 30.9% of cases because most of our patients presented late to the hospital with established complications which required emergency surgical intervention. Anorectal manometry was not performed in this study due to lack of this facility in our centre.

The principle of management of Hirschsprung’s disease is the removal of the aganglionic portion and a “pull-through” of the proximal ganglionated bowel [29], first described by Swenson and Bill in 1948 [14]. Later, Duhamel [15] and Soave [16] described the retrorectal pull-through and endorectal pull-through respectively. These three procedures have been widely used in the surgical management of this condition and the outcome and prognosis has been very good and comparable among the three procedures [14-16]. However, the choice of technique is still influenced by the training of the surgeon and the available resources and the presentation of the patient. In agreement with other studies done in developing countries [11,12], Swenson pull through was the most common surgical procedure performed in this study. Duhamel pull through which is commonly practiced by many surgeons in developed countries is not popular in resource- limited countries due to lack of GI stapler needed for the final anastomosis. This is reflected in our study where only 12.8% of patients underwent Duhamel pull through. Due to unavailability of GI stapler in our centre we have developed our own technique of hand anastomosis as described by Nasir *et al*. [12] in Nigeria. Traditionally, these pull through procedures have been done in three stages with the first stage being the creation of a diverting colostomy. The second stage performed much later when the nutritional status of the patient is improved (often between 6 and 12 months of the first surgery) involves the definitive pull-through while the final stage is the closure of the stoma [30]. This modality of management is associated with many problems including prolonged hospital stay; morbidity and mortality associated with colostomy in the neonate and infant, multiple exposures to anesthesia as well as increased cost of the management [29-32]. To avoid problems associated with staged procedures, attempts have been made by several workers to achieve a pull through without creating a stoma [29,31,32]. We have also shown in a previous study that colostomy is associated with high morbidity and a significant mortality in our children therefore a primary pull through where feasible would be ideal for our environment [33]. This will also translate to less financial burden as cost of hospitalization would be reduced. However, despite many problems associated with this treatment modality, many centers in developing countries still practice the traditional staged procedure for the treatment of Hirschsprung’s disease. This is reflected in our study where all the patients required preliminary colostomy as part of staged management. The reason for this is due to late presentation in the patients which makes a primary pull-through unsuitable because of the gross bowel distension. Also, many of the colostomies were done as a life-saving procedure in some of them as the conditions of the patients precluded any attempt at a primary extensive surgery. The advantages of a preliminary colostomy are numerous and include relief of obstruction, time for dilated colon to regain calibre and tone, making for easy colo-anal anastomosis as well as easy bowel washout prior to the pull-through. It may also help to reduce the incidence of postoperative enterocolitis [29]. Planning a staged procedure requires that the stoma be well sited in a biopsy-proven normal bowel [29,30]. However, this was not possible in our study due to lack of intra-operative frozen section facility as well as the emergency nature of the colostomy and therefore reliance had to be made in some cases on visual identification of the transition zone as well as presence of peristalsis on stimulation in order to site the stoma.

The majority of patients in the present study had short segment of aganglionosis localized to the recto-sigmoid region which is in keeping with other studies [11,12,29,30]. We could not establish the reason for this anatomical distribution.

The presence of complications has an impact on the final outcome of patients presenting with Hirschsprung’s disease. In this study, the complication rate was found to be 47.3%, a figure which is higher than that reported by other authors [11,12,29]. The reason for high rate of complications in this study may be attributed to late presentation as a result majority of patients to hospital in poor general condition.

The overall median duration of hospital stay following definitive pull through in the present study was 26 days which is higher than that reported by other authors [8,12]. This can be explained by the fact that the majority of patients who developed postoperative complications following pull through surgery stayed longer in the hospital. However, due to the poor socio-economic conditions in Tanzania, the duration of inpatient stay for our patients may be longer than expected.

The overall mortality rate in this study was 21.8% which is comparable with 23.8% reported by Nasir *et al*. [12] in Nigeria. The high mortality rate in this study was attributed to the age of patient at presentation (<4 weeks), late presentation of patients with established complications and presence of postoperative complications mainly sepsis. Addressing these factors responsible for high mortality in our patients is mandatory to be able to reduce mortality associated with this disease.

Normal continence and regular bowel emptying is the primary goal in the surgery for Hirschsprung’s disease [30]. During the follow up period, the majority of our patients had a good postoperative outcome (results) as more than 80% of patients (survivors) who had definitive pull through procedures had normal continence and regular bowel emptying. According to Nielson and Madsen classification [23], the results of Swenson’s and Duhamel’s pull through procedures were generally good in 87.8% and 42.9% of patients respectively. Similar observation was reported by Nazir *et al*. [12] in Nigeria. Soiling was a major late complication of both procedures observed during the follow up period. But it has been observed that the problem tended to lessen as the child grew older and with careful dietary selection. The incidence of faecal incontinence varies from less than 1% to as high as 32.7% [34-38]. In the present study, faecal incontinence was reported in only 2.1% of cases, a figure which is comparable to what was reported by Jarvi et al [37]. Although we do not have facilities such as anorectal manometry for measuring sphincter function in these patients clinical history and examination revealed that most of our patients are continent. The results of Soave’s procedures were poor in this study. However, since our duration of follow up was limited to only two years, we could not estimate the long term outcome of treatment.

The management of Hirschsprung’s disease poses many challenges to pediatricians and pediatric surgeons practicing in resource-limited countries like ours [8,10,12,21]. Ignorance, poverty, late presentation of patients with attendant complications, limited access to trained paediatric surgeons and limitation of facilities for prompt diagnosis are the hallmarks of the disease. Hence, multiple stages of management still predominate in sub Saharan Africa. A single stage pull-through in Africa would be indicated if patients present early without complications and the expertise is available to reduce morbidity of repeated surgery, cost to the parents and long waiting list time.

Delayed presentation and lack of frozen biopsy facility at our Centre to determine the level of aganglionosis were the major limitations in this study. However, despite these limitations, the study has provided local data that can be utilized by health care providers to plan for management guidelines for these patients. The challenges identified in the management of Hirschsprung’s disease in our environment need to be addressed, in order to deliver optimal care for these patients.

## Conclusion

In conclusion, while in most centers in the developed world, pull-through operations for Hirschsprung’s disease have evolved from the open technique through laparoscopically assisted pull-through to totally trans-anal pull techniques, late presentation in our setting militate against this. It is hoped that with early presentation and diagnosis, totally trans-anal pull techniques would also become routine in our patients. However, the open staged definitive pull through procedure still has a role to play in the management of Hirschsprung’s disease in our environment. Therefore, public health education is necessary with regard to seeking early medical attention. This should enhance early surgical correction and prevent fatal complications.

## Competing interests

The authors declare that they have no competing interests. The study had no external funding. Operational costs were met by authors.

## Authors’ contributions

JBM and PLC participated in study design, literature search, data analysis, manuscript writing and editing. In addition PLC submitted the manuscript. NMK, MM, ABC and PFR participated in data analysis, manuscript writing & editing. All the authors read and approved the final manuscript.
